# The founding of Zagreb's Institute for the Culture of Health: an important step toward a new medical paradigm

**DOI:** 10.3325/cmj.2015.56.1

**Published:** 2015-02

**Authors:** Veljko Đorđević, Marijana Braš, Slavko Kulić, Vida Demarin

**Affiliations:** 1 Center for Palliative Medicine, Medical Ethics and Communication Skills, University of Zagreb School of Medicine, Zagreb, Croatia veljko.djordjevic1@zg.t-com.hr; 2 Institute on World's Problems for Europe, Zagreb, Croatia; 3 Zagreb's Institute for the Culture of Health, Zagreb, Croatia

Health is a state of complete physical, mental, social, and spiritual well-being and not merely the absence of illness and exhaustion. It is one of the fundamental human freedoms: all intelligent beings and all vertebrates are entitled to it. It arises from necessity or will, but not from fear. The human species exercises this aspect of freedom through its way of life.

How can we accomplish such a health status of individuals and communities in the current social context? Can one grow and make progress outside of his or her context and culture? Does health exist only as a postulate of biology or is it something much larger? Are we able to focus on stimulating the healthy and creative forces in our population that are so important for coping with disease, maintaining hope, fighting for life, and not giving up?

We are witnessing an unprecedented development of personalized medicine. However, we must not lose sight of the patient as a person in favor of technological superiority (1). The medical interview provides a framework through which we can explore and understand patients' concerns, fears, misconceptions, and what they bring to their illness, while taking into consideration their culture, the availability of various treatment options, and financial considerations (2).

Medicine as a whole, particularly person-centered medicine, arises from the geobiosocial understanding of life. It is based on the principle of co-operation and tolerance in the evolution of life, and on the principle of co-operation in cell biology. This is opposite to the geobiopolitical understanding of life, based on conflict, confrontation with life and nature (which is the cause of all forms of illness – in humans, animals, and plants) (3). Modern pathology arises from the wrong types of knowledge and our mind’s incapacity to understand health and life. Good health is the product of both the individual and the community; health is not a given, it is something that must be fought for, worked toward, and invested in. It represents the ultimate material, spiritual, and cultural resource of a community. In order for this approach to be successful, we need to find ourselves on a path that leads to the humanization of man. We must stop living for illness and start fighting for health. We should view each person as a unique individual, whatever their biopsychosocial needs may be, and not merely as a diagnosis or a sum of their symptoms (4).

## Zagreb's Institute for the Culture of Health

Culture is central to the way we view, experience, and engage in all aspects of our lives and the world around us. Thus, even our definition of culture is shaped by the historical, political, and social contexts in which we live. Culture is an integrated pattern of human knowledge, beliefs, and behaviors that depends upon a capacity for learning, and the transfer of knowledge to succeeding generations (5). Although culture is widely accepted as a factor associated with health and behavior, its role in public health practice and research to date has been more rhetorical than applied. Efforts to eliminate health disparities must be informed by the influence of culture on the attitudes, beliefs, and practices. The main stakeholders in this process are public health policymakers and the health professionals responsible for the delivery of medical services and public health interventions (6). As a tool to promote the development of the culture of health, the Zagreb’s Institute for the Culture of Health (ZICH) was founded in 2013. The conceptual framework of the ZICH is devoted to education, organization, and research in several domains: the culture of health, person-centered medicine, people-centered health care, medical humanities, and communication in medicine. The interdisciplinary team involved in the creation of ZICH includes experts from almost all fields of medicine and health care. It started with a close co-operation with numerous national and international organizations and institutions in various educational and research activities. ZICH is globally one of the first institutionalized attempts to systematically promote the culture of health among professionals and the public.

A culture of health relies on strong teamwork, a subject of interest to ZICH. Within a health-service workplace, it is important to identify the competencies related to effective teamwork. A special point in ZICH's development was the Memorandum of Understanding with the Center for Palliative Medicine, Medical Ethics, and Communication Skills at the University of Zagreb School of Medicine (CEPAMET) at the end of 2014 (7). ZICH forms the link between those dealing with science, clinical work, and medical humanities. Additionally, emphasis is placed on the connection between experts and patient groups, where ZICH promotes the richness of exchange of various experiences around the world, appreciating various perspectives and transcultural differences. The collaboration is open to both national and international partners in order to further contribute globally to the development of the culture of health.

## Art and culture of health

When we talk about culture of health, we must never forget the importance of communication through art. Art is the most effective way to approach people and produce effects that no other means of communication can achieve. Since it is one of the most powerful tools in the education of health care professionals and general public about the culture of health, it is used as a therapeutic technique as well as a means of raising public awareness of culture of health (8). Art as a universal language is perhaps the most powerful means to connect with and improve people from all over the world. By influencing people's emotions, it forms an invaluable part of today's medicine. Creation is an act of survival, growth, and development of an individual as well as a community, and ZICH puts a special emphasis on a project entitled Neuroscience, Art, and the Culture of Health.

## On the front line of health

Throughout the Homeland War (1991-1995) and during its aftermath, a significant number of people were exposed to long-term and intense traumatic experiences. As scientists, physicians, and as humanists, we must ask ourselves how much we have done to improve health outcomes and social support of these people (9). Despite all of our knowledge regarding the link between chronic stress, posttraumatic stress disorder (PTSD), and physical health, we do not have clear epidemiological data regarding the physical health of war veterans and their family members as well as other victims of the Homeland War, nor do we have concrete programs related to their physical rehabilitation. In this context, the project entitled On the Front Line of Health was created. There is a lack of education of health care professionals regarding the specifics of communication with patients with PTSD as well as the link between PTSD and physical health. Those people are an important part of our community, and the worst approach would be to ignore them. We must act now to support those in need, and ZICH will do it in co-operation with other institutions and organizations.

## *Ars medica* and the culture of health

Patients are persons within their physical, psychological, social, and spiritual totality (10). Medical education is a critical component of the promotion of the culture of health, which requires a revised approach to the teaching of clinical and communication skills. It is the first step in the establishment of a culture of health among practitioners and patients alike. Education includes person-centered medicine, and it is the intervening variable in the development of a culture of health and medicine as a culture of life. It is important to maintain health as a natural condition. Attitude toward life and nature is something we learn. Education enables us to free our being from captivity in order to build a culture of health. The culture of health is communication, and communication is crucial for the implementation of the culture of health. The culture of health in its broadest sense is a cultivated behavior related to health and society. Along with the aforementioned activities, ZICH could significantly contribute to the development of the culture of health in Croatia and abroad. It is time for us to show everyone that we can learn and grow together. *Ars medica* is as much “art in medicine” as it is “the medicine of art.” It is a journey from a culture of illness toward a culture of health, from symptoms and diagnoses toward human beings and persons.
